# Disrupted topological organization of brain connectome in patients with chronic low back related leg pain and clinical correlations

**DOI:** 10.1038/s41598-025-91570-3

**Published:** 2025-03-04

**Authors:** Yuqi Ji, Xiao Liang, Yixiu Pei, Xiaoying Zuo, Yanyan Zhu, Jie Xu, Qinmei Kuang, Ziwei Yang, Fuqing Zhou, Yong Zhang

**Affiliations:** 1 Department of Radiology, the First Affiliated Hospital, Jiangxi Medical College, Nanchang University, Nanchang, 330006 Jiangxi Province China; 2 Jiangxi Province Medical Imaging Research Institute, Nanchang, 330006 Jiangxi Province China; 3 The Second Affiliated Hospital of Xi’an Jiaotong University, Xi’an, 710000 Shaanxi Province China; 4 Department of Pain Clinic, the First Affiliated Hospital, Jiangxi Medical College, Nanchang University, Nanchang, 330006 Jiangxi Province China

**Keywords:** Structural connectivity or connectome, Functional connectivity or connectome, Chronic low back-related leg pain, Mediation analysis, Spatial discrimination, Chronic pain, Sensory processing, Peripheral neuropathies

## Abstract

Chronic pain is associated with persistent alterations in brain structure and function. However, existing research has not fully explored the relationship between brain network topological properties and clinical symptoms in patients with chronic low back-related leg pain (cLBLP). In this study, we collected resting-state functional and structural magnetic resonance imaging data, along with clinical symptom evaluation data, from 32 cLBLP patients and 31 healthy controls. A large-scale complex network analysis was conducted to evaluate the global and nodal topological properties of functional and structural brain networks. Statistical analyses were performed to determine the associations between network properties and clinical variables. The results showed significant alterations in both global and nodal topological properties of functional and structural brain networks in cLBLP patients compared to healthy controls. Additionally, a direct correlation was found between structural network properties and spatial discrimination ability, measured by two-point tactile discrimination values, while no significant association was observed between functional connectivity and spatial discrimination. This study demonstrates that cLBLP patients exbibit a decreased local efficiency of functional connectivity network and increased compensatory global efficiency of structural connectivity network. Notably, alterations in the structural connectome, rather than the functional connectome, play a more significant role in deterioration of foot tactile spatial acuity in cLBLP patients.

*Trial registration*: This trial was registered in the Chinese Clinical Trial Registry with the registration number ChiCTR2200055321 on 2022-01-06.

**Supplementary Information:**

The online version contains supplementary material available at 10.1038/s41598-025-91570-3.

## Introduction

In recent epidemiological surveys, the prevalence of low back pain has been reported to be as high as approximately 70 ~ 80% (2016)^1^, and it has become one of the major causes of disease burden in China^2,3^. Several studies have investigate the distinct neuroplasticity and synaptic remodeling occurring in patients suffering from simple low back pain, including gray matter volume and density alterations^4,5^, white matter integrity^6,7^, functional activation^8^ and functional connectivity (FC) alterations^5,9^. Low back-related leg pain (LBLP) patients, as a specific subtype, often receive more attention than simple low back pain due to its higher rates of disability, lower quality of life and less postoperative remission rate^2,3^. However, the characteristic sciatica or referred leg pain is not sufficient to explain these phenomena or manifestations in patients with LBLP, especially those in the chronic phase.

Recent magnetic resonance imaging (MRI) studies have confirmed a significant reduction in DTI-derived fractional anisotropy (FA) values of the compressed nerves in LBLP patients, which closely correlates with their clinical symptoms^10^. Sciatica due to lumbar disk herniation can lead to specific local alterations in gray and white matter^11^, including reduced gray matter volume or cortical thickness in pain-related areas, increased gray matter volume in affective reaction regions, diffuse decreases white matter volume in the orbitofrontal cortex. Functional MRI (fMRI) studies have revealed disrupted within-network FC within the pain matrix and sensory processing areas among chronic LBLP (cLBLP) patients^12–15^, as well as lower cross-network FC between the salience network and ascending nociceptive pathway (Asc)^16^, with compensatory hyperactivity primarily observed in information processing area^13,14^. In addition, specific frequency bands have been found to exhibit distinct disease-related functional activity changes among cLBLP patients^13–16^. Therefore, selecting specific frequencies may better elucidate clinical relevance by capturing the dynamic alterations in plasticity or maladaptation associated with chronic pain, which underlie significant alterations in emotion, attention, and cognition in patients with cLBLP^16–18^.

The human brain is a highly flexible and adaptable network, capable of reconfiguring itself to compensate for clinical symptoms when the brain region is damaged, or functionally abnormal. For example, chronic nociceptive stimuli in chronic pain can lead to damage or plastic in brain structural or functional networks^7,11,19–21^. However, previous studies on LBLP or simple low back pain, structural changes in the brain did not match or even reverse functional brain changes^11–15,22^. Moreover, it remains unclear whether local brain regions with altered brain function or structure play a significant role or if their integration within network is more correlated with clinical variables, such as tactile spatial acuity. A meta-analysis of 19 recent studies has demonstrated that decreased tactile acuity is an important clinical manifestation of central plasticity in chronic low back pain. Furthermore, in patients with cLBLP, whether “structural connectome, functional connectome, and clinical variables” are directly associated or whether mediating relationships are required for association.

**Fig. 1:**
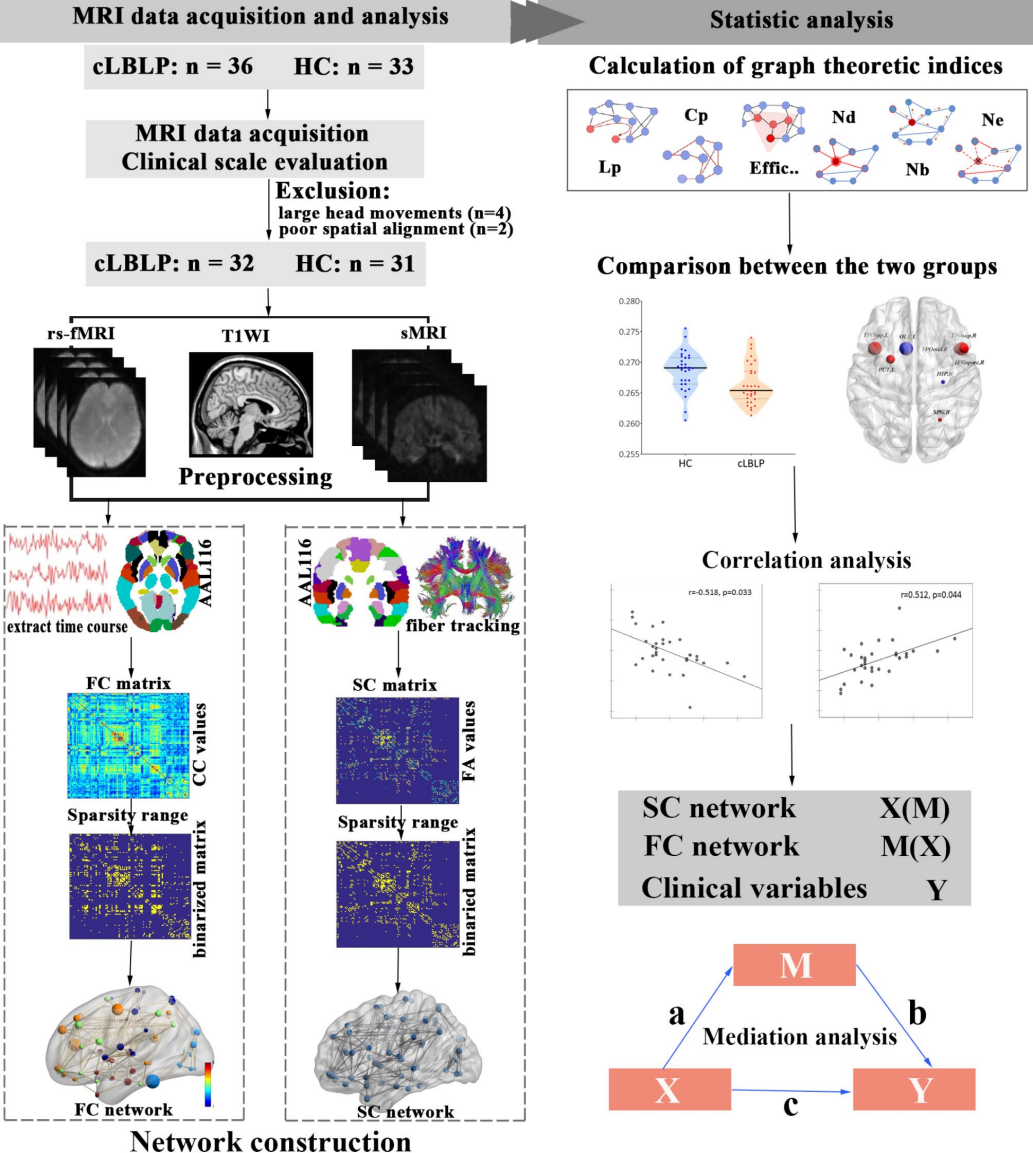
Flow chart of the study of topological properties of brain networks in patients with chronic low back-related leg pain (cLBLP). The study included the following steps: recruitment of subjects, data collection and preprocessing, construction of SC network and FC network, calculation of network properties and comparison between groups, correlation analysis and mediation analysis. Note: HC, healthy controls; Lp, characteristic path length; Cp, clustering coefficient; Effic, efficiency; Nb, nodal betweenness; Nd, nodal degree; Ne, nodal efficiency; X, independent variable; M, mediator variable; Y, dependent variable.

To address the aforementioned questions, we hypothesized that cLBLP patients exhibit damage or altered plasticity in the FC network or structural connectivity (SC) network, and that structural or functional connectomes play a mediating role in clinical relevance. In the present study (refer to Fig. 1 for the flow chart), we employed the graph-theory methods to capture abnormal FC network and SC network properties, as well as their associations with clinical symptoms in cLBLP patients. Second, we aimed to establish the mediating roles of these identified SC network metrics in explaining the relationship between FC network metrics and clinical symptom scores. Finally, we investigated frequency-specific contribution by analyzing FC network property within slow-4 and slow-5 frequency bands^23^. Exploring the connectivity properties of brain networks along with its clinical relevance could provide valuable macroscopic insights into neurobiological processes among cLBLP patients.

## Results

### Demographics and clinical evaluation

After exclusion of 4 cLBLP patients and 2 healthy controls (HCs) due to the large head movements (greater than 3.0° angular rotation or 2.0 mm translation) and poor spatial alignment, 32 cLBLP patients (19 males and 13 females) and 31 HCs (19 males and 12 females) were finally enrolled in the present study. There were no significant differences in age (*p* = 0.265) or sex (*p* = 0.289) between the two groups. The clinical variables for the two groups are reported in Table 1, including disease duration, visual analogue scale (VAS) score, Barthel index (BI) score, Japanese Orthopaedic Association (JOA) score, Fugl-meyer score, two-point tactile discrimination (2-PD). These data indicate that cLBLP patients have varying degrees of impairment in living, daily activities, and tactile sensory discrimination.

**Table 1:** Demographic and clinical variables of chronic LBLP patients and healthy controls.

Clinical variables	cLBLP patients (N = 32)	Healthy controls (N = 31)	p value
Demographic data			
Age, years	53.00 ± 8.14	51.32 ± 5.35	0.265
Sex, N	13F/19 M	12F/19 M	0.289
Clinical characteristics and scales			
Disease duration, month	33.31 ± 46.12	NA	NA
Visual analogue scales (VAS) score	5.78 ± 0.95	0	NA
Barthel index (BI) score	87.66 ± 11.98	NA	NA
Japanese orthopedic association back pain evaluation questionnaire score	13.94 ± 5.07	NA	NA
Daily activities	6.19 ± 3.26	NA	NA
Fugl-Meyer assessment	19.62 ± 2.38	NA	NA
Two-point tactile discrimination test (2-PD)			
2-PD test score of right hand	25.53 ± 5.73	NA	NA
2-PD test score of left hand	25.81 ± 5.79	NA	NA
2-PD test score of right foot	29.97 ± 7.87	NA	NA
2-PD test score of left foot	30.25 ± 5.91	NA	NA

*cLBLP* chronic low-back-related leg pain, *F* Female, *M* male, *NA* not applicable or not available.

### Altered global network metrics in the cLBLP patients

**Fig. 2:**
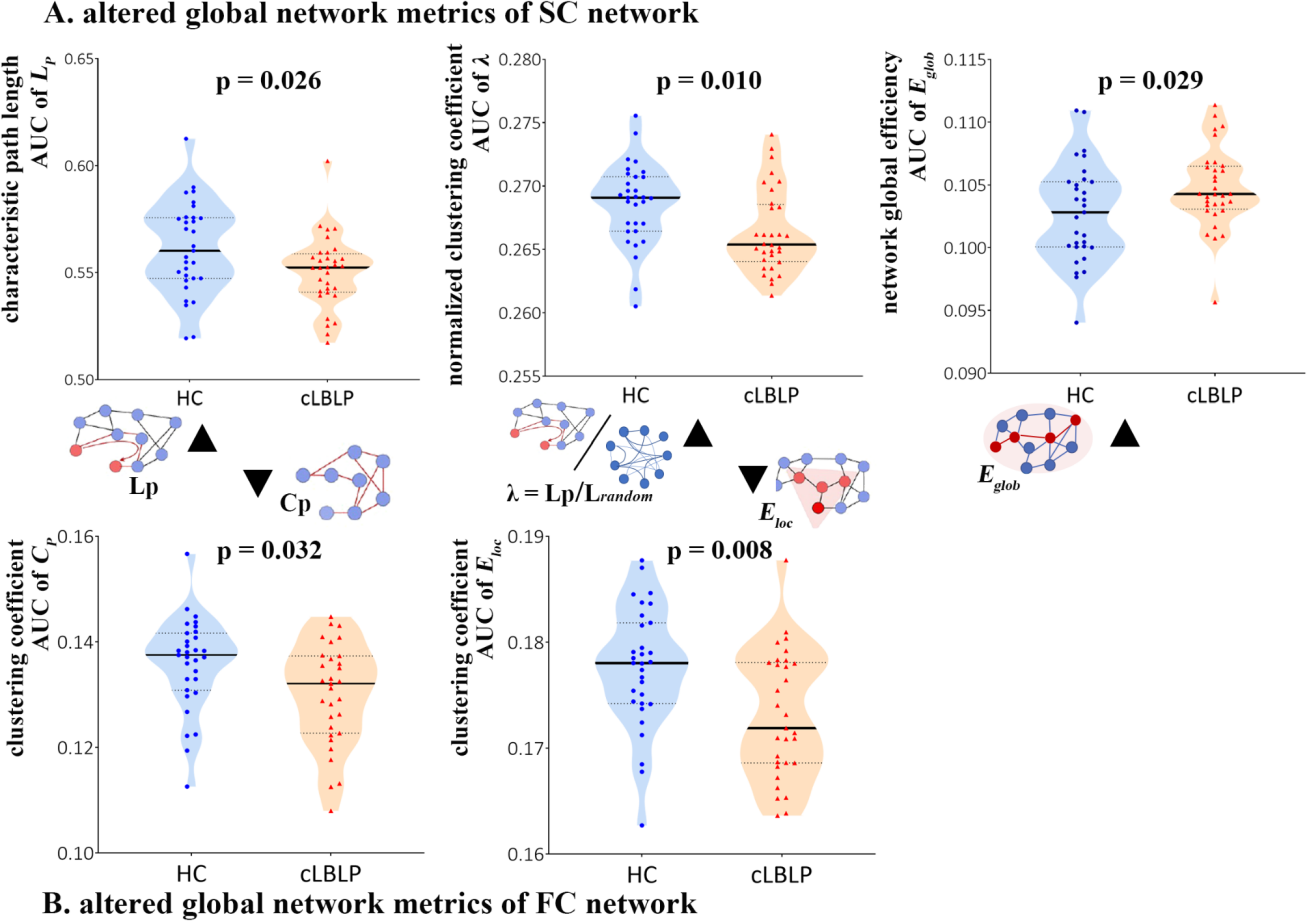
Group differences in the global network metrics of SC and FC network (*p* < 0.05, with Bonferroni correction). *Note*: (**A**) Significantly reduced characteristic path length, normalized clustering coefficient and increased network global efficiency of SC networks were observed in cLBLP patients relative to the controls. (**B**) Reduced local efficiency and clustering of FC networks in cLBLP patients relative to controls were found.

Both the structural connectome and functional connectomes of the cLBLP patients and HCs showed small-world properties (standardized characteristic path length (Lambda, $\:\lambda\:$) ≈ 1, standardized clustering coefficient (Gamma, $\:\gamma\:$), > 1, small-worldness (Sigma, $\:\sigma\:$) $= \gamma \div \lambda > 1$). For the structural connectome, cLBLP patients showed decreased characteristic path length, normalized characteristic path length and increased network global efficiency compared with the controls (all *p* < 0.05, with Bonferroni correction) (Fig. 2A and Table S1). For the functional connectome, cLBLP patients showed decreased local efficiency (*p* = 0.008, with Bonferroni correction) and clustering (*p* = 0.032, with Bonferroni correction) compared with the controls (Fig. 2B and Table S1).

### Altered nodal properties in the cLBLP patients

**Fig. 3:**
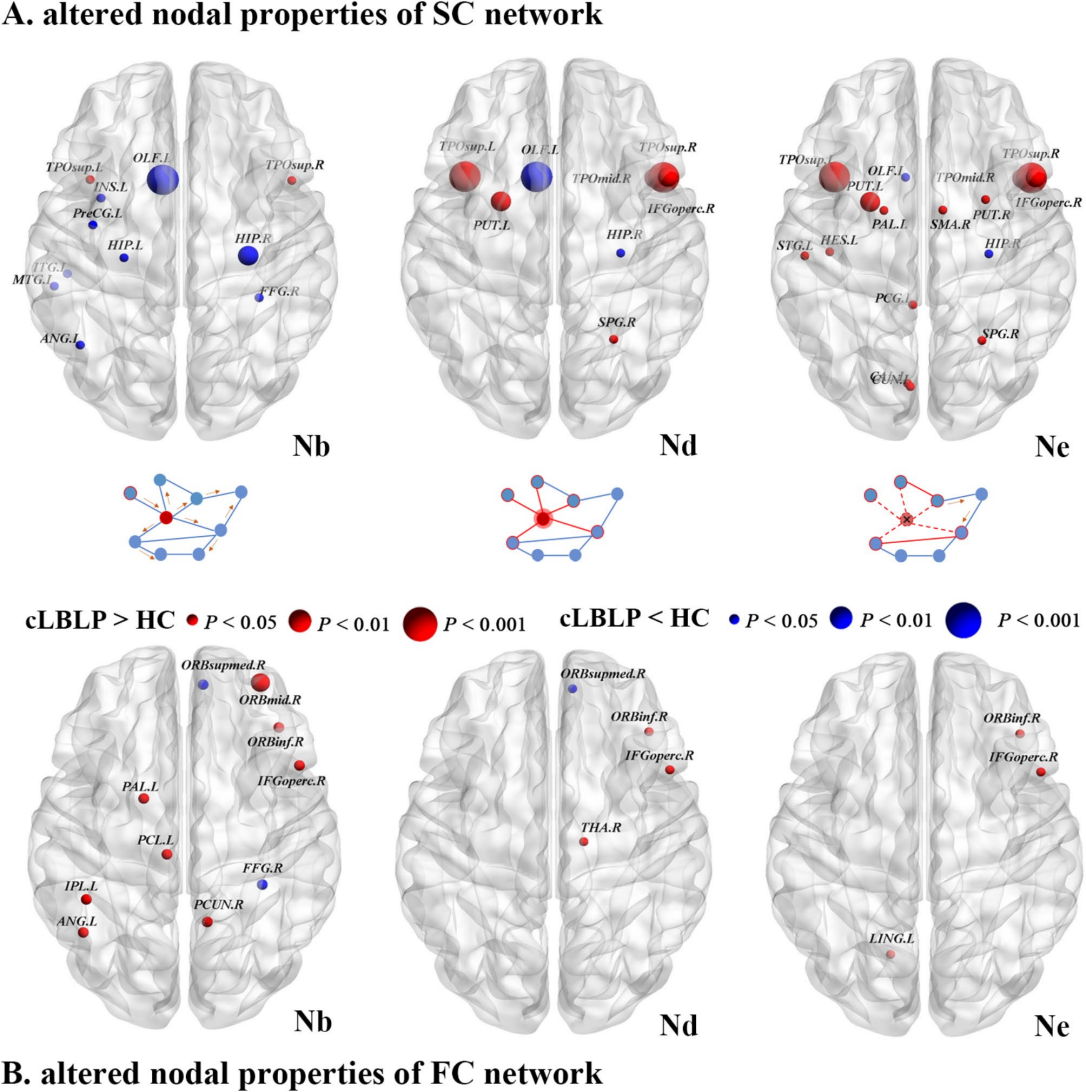
Distributed brain regions with significant alterations in the nodal properties of SC and FC network between cLBLP patients and healthy subjects (*p* < 0.05, with Bonferroni correction).

For the structural connectome, cLBLP patients exhibited increased nodal betweenness (Nb) in the bilateral superior temporal gyrus (TPOsup), while experiencing decreased Nb in the left insula (INS), left middle temporal gyrus (MTG), bilateral hippocampus (HIP), etc. Additionally, they demonstrated increased nodal degree (Nd) in the bilateral temporo-parieto-occipital (TPO) and putamen (PUT), but decreased Nd in the left olfactory (OLF) and left HIP. Furthermore, there was an increase in nodal efficiency (Ne) observed in the bilateral TPO, PUT and left pallidum (PAL), as well as right supplementary motor area (SMA), etc. These finding are depicted in Fig. 3A and Table S2.

Regarding the functional connectome presented in Fig. 3B and Table S3, cLBLP patients displayed several notable alterations: (1) increased Nb was observed within various regions such as the right middle frontal orbital gyrus (ORBmed), right inferior frontal orbital gyrus (ORBinf), right inferior frontal gyrus (IFG), the left paracentral lobule (PCL), and left angular gyrus (ANG), but decreased Nb in the right medial orbital superior frontal gyrus (ORBsup) and Fusiform gyrus (FFG); (2) increased Nd was found in the right ORBinf, right inferior frontal gyrus (IFGoperc) and right thalamus (THA), but decreased Nd in the right ORBsup; (3) increased Ne was detected in the left lingual gyrus (LING), right ORBinf, as well as in the right IFGoperc.

### Relationship between network metrics and clinical variables

**Fig. 4:**
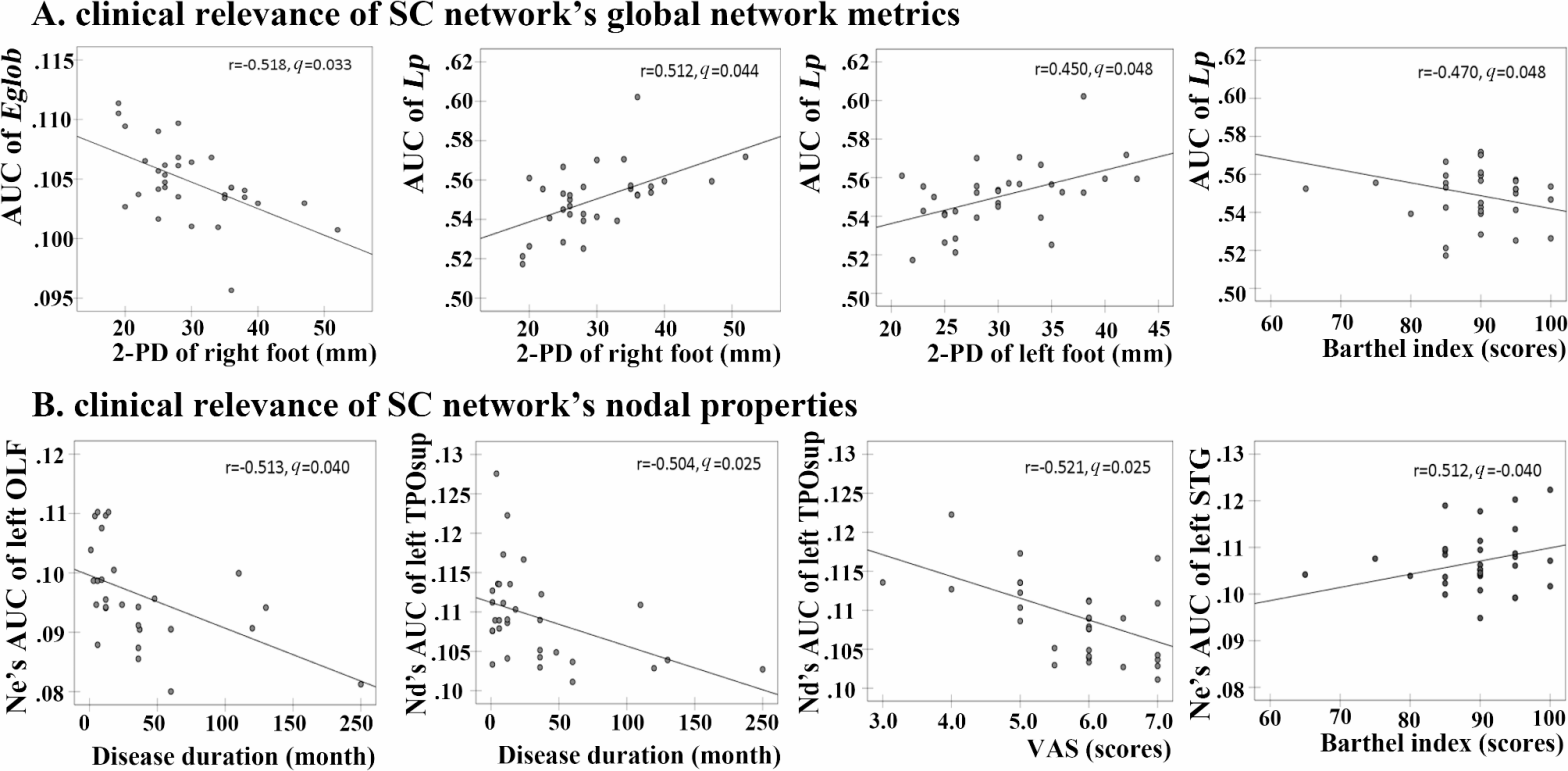
Correlations between network metrics and clinical assessments in patients with cLBLP (Benjamini-Hochberg false discovery rate correction *q* value < 0.05). *Note*: 2-PD, two-point tactile discrimination test; AUC, area under curve; E_glob_, network global efficiency; OLF, olfactory cortex; TPOsup, superior temporal gyrus; STG, superior temporal gyrus; VAS, visual analogue scale; L_p_, characteristic path length.

Regarding the clinical relevance of cLBLP patients, significant correlations were observed between the global and node properties of SC network, as well as the node properties of FC network, with various clinical parameters (*p* < 0.05, Figure S1 and Figure S2). However, no correlation was found between the global properties of FC network and clinical parameters (*p* > 0.05, Figure S2). However, after applying false discovery rate (FDR) correction, only significant clinical correlations were identified in the structural connectome but not in the functional connectome (Fig. 4).

The altered global metrics of SC network revealed the following correlations: (1) a negative correlation between the area under the curve (AUC) value of $\:{E}_{glob}$ and the scores of the right foot 2-PD test (FDR *q* = 0.033); (2) a negative correlation between the AUC value of $\:{L}_{p}$ and BI scores (FDR *q* = 0.048), and (3) a positive correlation between the AUC value of $\:{L}_{p}$ and both left foot 2-PD (FDR *q* = 0.048) and right foot 2-PD test (FDR *q* = 0.044).

In terms of altered nodal properties, the Nd’s AUC of left TPOsup exhibited negatively correlations with VAS score (FDR *q* = 0.025) and disease duration (FDR *q* = 0.025) among cLBLP patients; the Ne’s AUC of left OLF displayed a negative correlation with disease duration (FDR *q* = 0.040); the Ne’s AUC of left STG demonstrated a positive correlation with the BI scores (FDR *q* = 0.040).

### Mediation analysis of network topological properties with clinical variables

After conducting correlation analysis, we selected relevant “structural connectome-functional connectome-clinical variables” pairs for further mediation analysis in order to examine whether the functional connectome was mediated through the structural connectome and clinical variable. We observed significant regression effects between multiple nodal properties of structural connectome and clinical variables (*p*_b_ < 0.05), indicating a direct association (Table 2). However, this study did not find evidence supporting the mediation of SC network properties in the relationship between FC network properties and clinical variables, or did it find evidence supporting the mediation of FC network properties in the relationship between SC network properties and clinical variables.

**Table 2:** Test results of mediation models for Sequential regression and Bootstrap sampling methods in nodal properties of SC network and FC network with clinical variables.

Mediation model	Total effect: c (p)	Direct effect: c’(p)	Impact of X on M: a (p)		Impact of M on Y: b (p)	Indirect effect (a*b)	a*b (95% BootCI)	Conclusion (mediating effect)
Altered Nb of SC and FC network								
X = Nb’s AUC of right ORBinf (FC network)	-0.080 (0.309)	-0.045 (0.456)	-0.081 (0.442)	0.437 (0.019)*		-0.035	-0.244 ~ 0.063	Non-significant
M = Nb’s AUC of left TPOsup (SC network)								
Y = 2-PD of right foot								
X = Nb’s AUC of right ORBinf (FC network)	0.038 (0.587)	0.063 (0.368)	-0.081 (0.442)	0.318 (0.028)*		-0.026	-0.277 ~ 0.066	Non-significant
M = Nb’s AUC of left TPOsup (SC network)								
Y = 2-PD of left foot								
Altered Nd of SC and FC network								
X = Nd’s AUC of right IFGoperc (FC network)	0.216 (0.271)	0.194 (0.273)	-0.018 (0.838)	-1.180 (0.024)*		0.022	-0.122 ~ 0.210	Non-significant
M = Nd’s AUC of right TPOmid (SC network)								
Y = Fugl-Meyer scores								
Altered Ne of SC and FC network								
X = Ne’s AUC of left LING (FC network)	85.899 (0.603)	73.558 (0.677)	-0.038 (0.742)	-321.667 (0.028)*		12.341	-0.137 ~ 0.135	Non-significant
M = Ne’s AUC of right SPG (SC network)								
Y = 2-PD of right foot								
X = Ne’s AUC of left LING (FC network)	85.899 (0.603)	56.755 (0.795)	-0.078 (0.534)	-373.348 (0.011)*		29.145	-0.099 ~ 0.148	Non-significant
M = Ne’s AUC of right TPOsup (SC network)								
Y = 2-PD of right foot								

*p < 0.05 in regression equations. *X* independent variable, *M* mediator variable, *Y* dependent variable, a*b(95%BootCI), the 95% confidence interval of the indirect effect value, which does not include 0, indicates that there is an mediant effect, otherwise there is no mediating effect. *ORBinf* superior frontal gyrus (orbital part), *TPOsup* superior temporal gyrus, *IFGoperc* inferior frontal gyrus (opercular part), *TPOmid* middle temporal gyrus, *SPG* superior parietal gyrus.

### Sub-frequency band FC network properties alterations of cLBLP patients

**Fig. 5:**
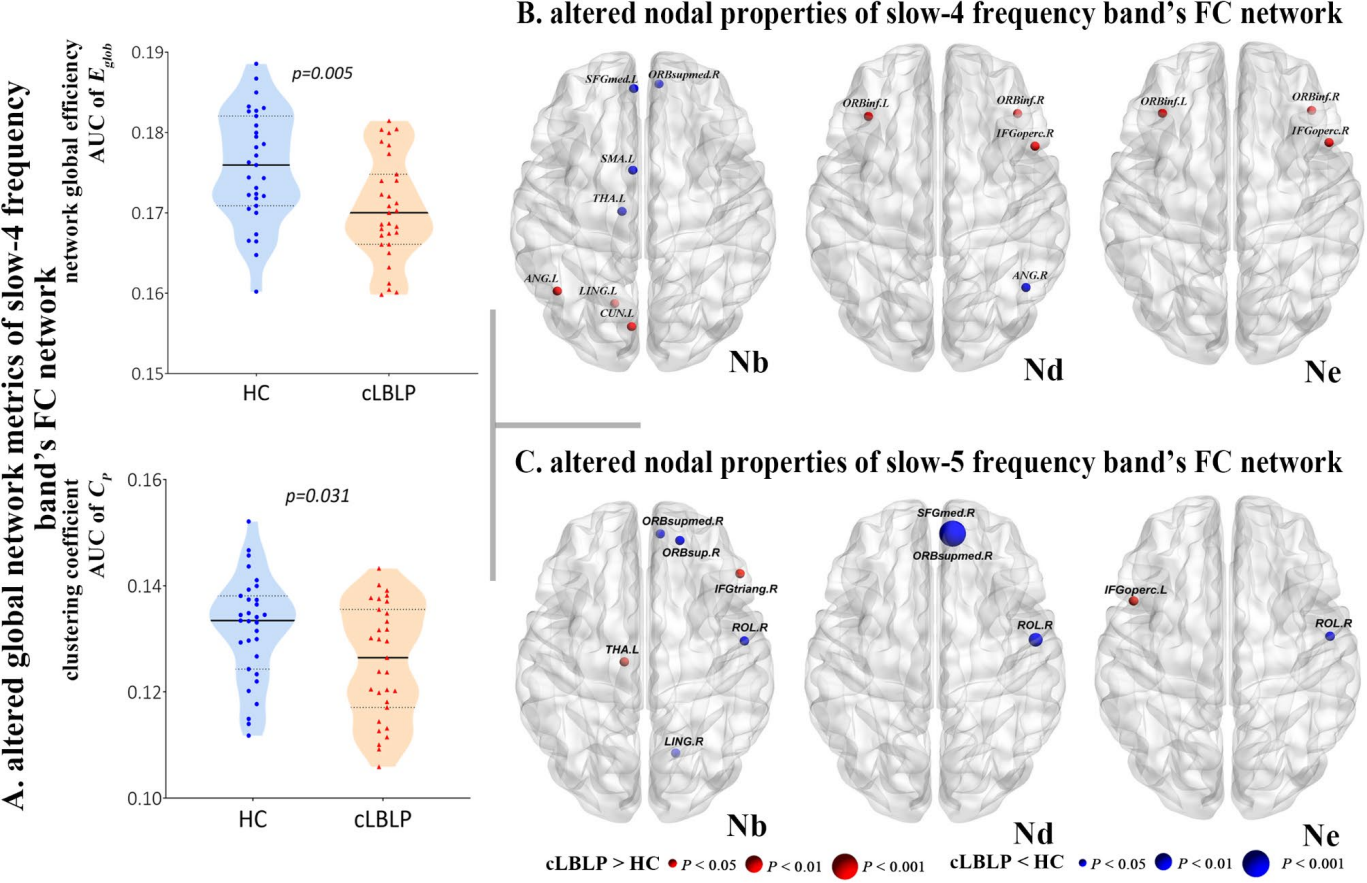
Group differences in the global network (**A**) and nodal properties (**B**, **C**) of sub-frequency band functional connectome between cLBLP patients and healthy subjects (*p* < 0.05, with Bonferroni correction). * Note*: In cLBLP patients, the functional connectomes of slow-4 frequency band exhibited a significant reduction in clustering coefficient and global efficiency compared to the controls (**A**). Furthermore, altered nodal properties were observed in both slow-4 (**B**) and slow-5 (**C**) frequency band’s functional connectomes in cLBLP patients relative to controls.

For the functional connectome in the slow-4 frequency band, cLBLP patients exhibited a significant decrease in local efficiency (*p* = 0.005, with Bonferroni correction) and clustering (*p* = 0.031, with Bonferroni correction) compared with the controls (Fig. 5A and Table S1). However, there was no significant difference in global properties of functional connectome in the slow-5 frequency band between cLBLP patients and controls (Table S1).

In the nodal properties of slow-4 frequency band’s functional connectome, cLBLP patients showed the following differences (Fig. 5B and Table S4): (1) increased Nb were observed in the left ANG, left CUN and left LING, and decreased Nb were shown in the right ORBsupmed and left SFGmed, left THA, and left SMA; (2) decreased Nd was shown in the right ANG, and increased Nd were shown in the bilateral ORBinf and right IFGoperc; (3) increased Ne were shown in the bilateral ORBinf and right IFGoperc.

In the nodal properties of slow-5 frequency band’s functional connectome, cLBLP patients showed the following differences (Fig. 5C and Table S5): (1) decreased Nb were distributed in the right ORBsup, right ROL, and right LING, and increased Nb was shown in the right IFGtriang and left THA; (2) decreased Nd were shown in the right ORBsupmed, right SFGmed, and right ROL; (3) increased Ne were shown in the left IFGoperc and right ROL.

The global properties of the functional connectome in the slow-4 and slow-5 frequency bands showed no significant correlation with clinical variables (*p* < 0.05). However, certain trends were observed between altered node properties and clinical variables (refer to Figure S3), which remained uncorrected after applying FDR correction.

## Discussion

In this study, we conducted a graph theoretical analysis to investigate the topological alterations of both structural or functional connectome in cLBLP patients. Our main findings were as follows: (1) cLBLP patients exhibit compensation in the structural connectome, which is manifested as shorter characteristic path ($\:{L}_{P}$), faster global transmission speed of information, and higher global efficiency ($\:{E}_{glob}$). However, this compensation decreases with the deterioration of tactile spatial acuity of limbs (2-PD). Moreover, increased disease duration and persistent pain further impair this compensation. (2) Although cLBLP patients have mild node compensation in the functional connectome, but the clustering coefficient of the global network decreases and the local information transmission efficiency ($\:{E}_{loc}$) decreases. (3) There is no mediating relationship among “structural connectome-functional connectome-clinical variables”, but direct association exist between multiple nodes properties of the SC network (such as TPOsup) attributes and clinical variables (2-PD values) in cLBLP patients. This finding highlight that structural connectome play a more crucial role than functional connectome in contributing to clinical manifestations such as deterioration of foot tactile spatial acuity in patients with cLBLP.

### Decreased local efficiency of FC network and tendency clinical relevance in cLBLP patients

In this study, we found that cLBLP patients have decreased local efficiency and clustering coefficient at the global level. However, at the node level, in addition to the decreased node properties (Nb and Nd) of default mode network (DMN)and visual association network, we also found increased node properties in multiple brain networks including fronto-parietal network. In addition to the typical features of low back pain and radiating pain in the lower limbs caused by nerve root compression, cLBLP patients also have impaired tactile spatial sensitivity and decreased ability to carry out daily activities, which seriously affect the daily work and living life of patients^24,25^. These symptoms are attributed to persistent pain and numbness signals that continuously input into the brain, leading to abnormal sensory and pain system information processing in the central nervous system of cLBLP patients. Consequently, these may interfere with other conscious and unconscious processes, resulting in unpleasant experience and even cognitive impairment^26^. The alterations in local neuroplasticity caused by abnormal information processing can disrupt the balance between inhibition and excitation through the functional connectivity of local neurotransmitters, thereby causing distributed changes in functional connectivity among cLBLP patients. Previous studies have confirmed that cLBLP patients have alteration in brain activity in pain matrix, sensory-processing regions and the default-mode network^13–16^. Furthermore, dysfunction of cross-network communication has also been demonstrated among patients with cLBLP^16^. These findings support our results indicating decreased local efficiency among patients with cLBLP.

Moreover, a trend towards correlation between the altered FC network properties and clinical variables was observed; however, this trend could not be corrected for FDR. This finding suggests that there is significant heterogeneity regarding functional connectome alterations among patients with cLBLP.

Considering the potential frequency-dependent nature of functional alteration of cLBLP patients^16^, this study further investigated the FC network topological properties within two sub-frequency bands. The results revealed more pronounced alterations in FC network topological properties within slow-5 frequency band compared to the slow-4 frequency band, indicating distinct mechanisms and physiological functions associated with different oscillatory bands in the brain^27^. Such characterizations have been demonstrated in several studies, particularly in the adjacent Slow5 and Slow4 bands, which have been found to either compete or coordinate with each other in certain cases; however, the specific mechanisms remain unclear.

### Progressively decompensated SC network properties in cLBLP patients

Multiple functional systems are interconnected through white matter fiber tracts, forming a brain network that facilitates information transmission. White matter fiber tracts serve as the highway of functional network and play a crucial role in facilitating information exchange within functional brain regions. In the present study, we observed that patients with cLBLP exhibited higher global efficiency of SC network, which was negatively correlated with tactile spatial acuity of the right foot. This finding suggests that in cLBLP patients with decreased tactile spatial acuity, the degree of increase in global efficiency in patients was not as pronounced as in patients with good tactile spatial acuity. Furthermore, our correlation analysis of the efficiency of nodes (OLF and STG) indicates that cLBLP patients may gradually lose compensatory mechanisms within the structural connectome as disease duration and severity worsen. Previous studies have reported structural alterations in the frontal, temporal and insular lobes among individuals experiencing chronic pain^7,28^. For example, patients with chronic low back pain have demonstrated localized reductions in subcortical white matter adjacent to the left prefrontal cortex, right premotor cortex, and the left anterior limb of the internal capsule^11^. These alterations highlight both neuronal damages resulting from persistent pain stimulation and neuroplasticity mechanisms employed by the brain to compensate for chronic pain^29^. The observed alterations in cerebral white matter structure provide a basis for understanding distributed alterations within functional connectome among patients with cLBLP.

During the chronic course of the disease, patients with high pain sensitivity in cLBLP exhibit a slow transmission of information. This suggests a potential early stage or absence of severe symptoms where neural plasticity’s protective function against loss may be compromised. Furthermore, our correlation analysis revealed that cLBLP patients with longer disease duration, higher pain intensity, and poorer tactile spatial sensitivity also exhibited slower information transfer, indicating a potential loss of neuroplasticity’s protective function in maintaining chronicity in LBLP patients^30^.

Chronic back pain, including cLBLP, is known to be associated with altered tactile acuity, resulting in higher 2-PD values compared with HC^31^. There was no significant difference in 2-PD values between affected and unaffected sites, further confirming that the test of tactile spatial acuity can serve as a cortical representation of tactile perception^32,33^. Neuroplasticity in S1 is believed to be associated with deficits in tactile acuity in patients with chronic back pain. Moreover, improvement in tactile acuity have been linked to corresponding changes in somatosensory processing despite treatment such as acupuncture^34,35^. This study provides additional support for the role of structural connectome alterations in influencing tactile spatial acuity by demonstrating a direct correlation between global properties of the structural connectome and 2-PD values among patients with cLBLP. Interestingly, this association does not necessarily require mediation through the functional connectome. This finding aligns well with our traditional understanding that “the brain structural network provides the physical basis, and the brain functional network is the direct reflection of the neuronal activity pattern”^36–38^. The reason behind this may be related to adaptive compensation involving cortical thickening^5,11^ and decreased functional connectivity^13,39^ within sensory processing areas during the chronic process experienced by LBLP patients.

The findings of this study suggest that cLBLP patients can effectively regulate the overall brain function by flexibly switching between different information processing modes, such as global integration and local separation, to adapt to structural damage or plasticity changes caused by the disease^7^. Consistent with other research^40–44^, the insights gained from mapping intrinsic brain connectivity networks provide a potentially mechanistic framework for understanding various aspects of human behavior.

There are several limitations of this study. Firstly, the inclusion of acute LBLP patients for comparison was lacking, as only cLBLP patients were included in this study. Including both acute and chronic LBLP patients in future studies may provide a more comprehensive understanding of how disease duration and VAS degree influence changes in brain network attributes. Secondly, although the patients specifically included in this study did not receive prescriptive medications or conservative treatment, it is important to note that cLBLP patients may self-administer analgesics to alleviate pain, however, the potential impact of irregular drug on brain function remain unknown. Thirdly, the study did not assess the mental and cognitive status of the cLBLP patients. Finally, variations in segmentation templates or network nodes could potentially affect the calculation and comparison of network topology properties among cLBLP patient. Furthermore, further investigation is needed to explore the causal relationship between altered brain network nodes in cLBLP patients and their association with clinical scales.

In conclusion, the present study demonstrates novel findings indicating that cLBLP patients exbibit a decreased local efficiency of FC network and increased compensatory global efficiency of SC network. It is suggested that alterations in the structural connectome, rather than the functional connectome, play a more significant role in the clinical manifestations observed in cLBLP patients, such as deterioration of foot tactile spatial acuity. These findings contribute to a more comprehensive understanding of the central mechanisms underlying cLBLP and provide new insights for future treatment strategies.

## Methods

### Participants

The inclusion criteria for patients with cLBLP are as follows: (1) an age within the range of 35–65 years; (2) definitive diagnosis of lumbar disc bulge or herniation combined with nerve root compression on imaging; (3) VAS score > 3; (4) lower back pain with radiation or involvement of the lower extremities; (5) disease duration of at least three months; and (6) ineffective prior conservative treatment with medications, e.g., anti-inflammatory drugs (Motrin, Advil and Naproxen) and acetaminophen (e.g., Tylenol) without opioids, exercise and physical therapy. The exclusion criteria included the following: (1) a history of other diseases or surgeries on the spine (deformity, trauma, infection, or tumour); (2) history of intracranial lesion or surgery; (3) history of other significant somatic or systemic diseases; and (4) MRI contraindications (cardiac pacemakers, metal dentures, etc.). After advertising recruitment, all HCs were screened using the Clinical Diagnostic Interview Nonpatient Version, and there were no significant cognitive impairments, head trauma, or MRI contraindications. In accordance with the above criteria, 36 cLBLP patients and 33 HCs were finally included in this study, and they were all right-handed.

This study is a prospective study. This study adhered to the principles outlined in the Declaration of Helsinki, and approval for the study protocol was granted by the Medical Ethics Committee of the First Affiliated Hospital of Nanchang University [Granted No: (2022) CDYFYYK (08–009)]. Prior to participation, everyone provided written informed consent.

### Clinical data acquisition

Initially, the disease course and onset of the patients with cLBLP were recorded. Afterwards, each cLBLP patient completed a series of clinical evaluation, including: (1) the JOA lower back pain assessment questionnaire, which serves as a crucial tool for evaluating the severity of low back pain, scored from − 6 to 29 points; higher scores indicate less dysfunction. This questionnaire specifically includes inquiries regarding subjective symptoms, clinical signs, and daily activities; (2) the VAS was employed to assess pain severity on a scale of 0–10, with higher scores reflecting greater pain severity; (3) the Fugl-Meyer scores were used to assess the severity of the pain; (4) the BI was used to assess the patients’ functional status in daily activities, scored from 0 to 100 points, where lower scores signify reduced ability in daily activities; and (5) the 2-PD test was used to evaluate tactile spatial discrimination in the bilateral fingertips and dorsum pedis, with a fingertip range of 3 to 6 mm and a dorsal range of 30 mm.

### MRI data acquisition

All participants underwent MRI scans on a 3.0 T MRI scanner (Trio Tim, Siemens, Munich, Germany) with standard eight-channel head coils. During the scan, all subjects were asked to keep their eyes closed, awake and calm. They were also given noise-cancelling headphones to minimize noise interference and control involuntary head movements, respectively. We acquired images with high-resolution 3D-T_1_-weighted imaging (3D-T_1_WI) sequences (repetition time/echo time (TR/TE) = 1900 ms/2.26 ms, field of view = 250 × 250 mm^2^, voxel size = 1.0 mm × 1.0 mm × 1.0 mm, slice thickness = 1 mm, 30 slices), resting-state functional MRI (rs-fMRI) sequences (TR/TE = 2000 ms/30 ms, field of view = 210 × 210 mm^2^, voxel size = 3.3 mm × 3.3 mm × 4.0 mm, slice thickness = 4 mm, 30 slices), and diffusion tensor imaging (DTI) sequences (TR/TE = 7700 ms / 104 ms, field of view = 234 × 234 mm^2^, voxel size = 1.8 mm × 1.8 mm × 2.0 mm, slice thickness = 2 mm, 62 slices).

### Imaging data analysis

#### Preprocessing of rs-fMRI data

The rs-fMRI data were preprocessing using Data Processing & Analysis for Brain Imaging Assistant (version 5.4, http://rfmri.org/DPABI) runing in MATLAB 2021b (Math Works, https://www.mathworks.com/products/MATLAB.html). The main steps included: data format conversion (DICOM to NIFTI), removal of the first 10 time points, slice timing and head motion correction, registration of T1 images to echo planar imaging (EPI) images, segmentation of T1 images by using the Diffeomorphic Anatomical Registration Through Exponentiated Lie Algebra (DARTEL) toolkit, spatial normalization and conversion to Monterey Neurological Institute (MNI) space, resampling (spatial voxel size of 3 × 3 × 3 mm^3^) and spatial smoothing (full width at half maxima of 6 mm), and linear detrending and regression of noisy signals (white matter, cerebrospinal fluid, head motion parameters with Friston-24 model). Finally, to diminish the influence of low-frequency drift and high-frequency physiological noise, we applied typical temporal bandpass filtering (0.01–0.1 Hz).

Previous studies have demonstrated that specific lower frequency bands (Slow5 and Slow4) can detect brain activity with more sensitivity^45,46^. Therefore, we performed temporal filtering on the fMRI data and completed the above steps for the Slow-5 (0.01–0.027 Hz) and Slow-4 (0.027–0.073 Hz) frequency bands.

#### Preprocessing of DTI data

We used PANDA software (http://www.nitrc.org/projects/panda/) and MATLAB, installed on a Linux operating system, to preprocess the DTI data. The main steps were as follows: conversion of data (DICOM to NIFTI), skull removal using BET, correction of eddy/head motion, calculation of FA using DTIFIT, fibre tracing using deterministic fibre tractography technique based on the fibre assignment by continuous tracking (FACT) algorithm, with propagation terminated at an angle greater than 45° or FA < 0.2, and registration to the Montreal Neurological Institute (MNI) space (voxel sizes of 2 × 2 × 2 mm^3^).

#### Network construction

The SC and FC networks were constructed using PANDA and GRETNA software (https://www.nitrc.org/projects/gretna), respectively, to reflect the physical connections between neurons and synapses (structural connectome) and the intrinsic neuronal activity patterns (functional connectome) of cLBLP patients. The nodes and edges are the key elements of each brain network. (1) Nodes were defined as follows: taking the Automated Anatomical Labelling Atlas (AAL116) as a template, we defined each brain region as a node of a FC and SC network, which consisted of 90 cortical/subcortical regions and 26 cerebellum regions. (2) Edges were defined as follows: we defined the Pearson correlation coefficients between the mean time series of node pairs as the unweighted edges for the FC network and the mean fractional anisotropy values of connected pathways between the two brain regions as the unweighted edge for the SC network. Afterwards, the undirected and symmetrical 116 × 116 matrix of FC networks and SC network for each subject was obtained.

#### Network topological properties analysis

According to previous studies^47^, setting a wide range of sparsity value, (0.1 ~ 0.34, with an interval step of 0.01)^48^ to calculate the AUC over the sparsity thresholds for global and nodal topological properties of the SC and FC networks, to avoid potential bias of any arbitrary single threshold selection.

The global small-world metrics are first discussed. The clustering coefficient (Cp) serves as a local feature of the brain network, reflecting its efficiency in local information processing. In contrast, the characteristic path length (Lp) represents the global characteristics of the network and indicates the overall efficiency of information integration among different brain regions. The standardized clustering coefficient ($\:\gamma\:$) and the standardized shortest path length ($\:\lambda\:$) are defined as the ratios of Cp and Lp to their corresponding values in a random network^49^, respectively. Together, these metrics define the small-world parameters. When the small-world scalar sigma$= \gamma \div \lambda > 1$, and both lambda ≈ 1 and gamma > 1 are satisfied simultaneously, it indicates that the brain network functions as a small-world network^44^. Next, we consider the global network efficiency metrics, which include global efficiency (Eg) and local efficiency (Eloc). The former refers to the parallel information transmission capability of the entire network, while the latter pertains to the information transmission capability among a single node’s neighbors. In addition to these global parameters, three nodal-level centrality parameters are examined: Nd, which is the number of nodes directly connected to a particular node; Nb, which measures the number of shortest paths between all other pairs of nodes that pass through this node; and Ne, which refers to the information transmission capability of each node within the network.

### Statistical analysis

After testing for normality of age and sex in the cLBLP and HC groups, two-sample t tests and chi-square tests were used to compare age and sex between the two groups (*p* < 0.05, two tailed). Descriptive clinical data (disease duration, BI, JOA, Daily Activities, VAS, Fugl-Meyer, 2-PD) were expressed as the mean ± standard deviation. All statistical analyses were performed using SPSS 21.0 software (IBM Corp, NY, USA, https://www.ibm.com/products/spss-statistics).

Each AUC of the global and nodal topological metrics were compared nonparametric permutation test (10,000 permutations) for between-groups differences, and the Benjamini-Hochberg false discovery rate (FDR q value < 0.05) correction was used for multiple comparisons, with age and sex as covariates^50,51^.

Partial correlation analyses were performed between the altered topological metrics and clinical variables with age and sex as covariates, with FDR correction q value < 0.05.

It is generally believed that the SC network is relatively stable and constitutes the physical basis of brain information processing, while the functional connectome disorder may be related to the dysfunction of disease^52^. Therefore, in this study, based on the results of the above partial correlation analyses, we set the shared relevant clinical scale as the dependent variable (Y), when the SC network topology metrics set as the independent variable (X), the FC network topological metrics set as the mediator variable (M), with age and sex as covariates. Conversely, FC network topological metrics is set as the independent variable (X), the SC network topological metrics set as the mediator variable (M). Initially, the sequential regression test was conducted on SPSS21 software for each model, and the significance of the three regression equations was successively obtained as follows: (1) X→Y: significance (*p*) of the direct coefficient c, (2) X→M: significance (*p*) of the coefficient a, and (3) X, M→Y: significance (*p*) of the coefficients c` and b. Considering the small sample size (*n* = 32) of this study, we decided to use the bootstrap sampling method implemented in SPSSAU (https://spssau.com/). We obtained several parameters, including total effect value (c), direct effect value (c`), indirect effect value (a*b), and 95% confidence interval for the indirect effect value (a*b 95% CI).

## Electronic supplementary material

Below is the link to the electronic supplementary material.

Supplementary Material 1

Supplementary Material 2

Supplementary Material 3

Supplementary Material 4

## Data Availability

The datasets used and or analysed during the current study available from the corresponding author on reasonable request.
