# Supplementary figures and images for: Disrupted topological organization of brain connectome in patients with chronic low back related leg pain and clinical correlations
Source: Sci Rep. 2025 Mar 4;15:7515. doi: 10.1038/s41598-025-91570-3 (PMC11876659; doi:10.1038/s41598-025-91570-3)

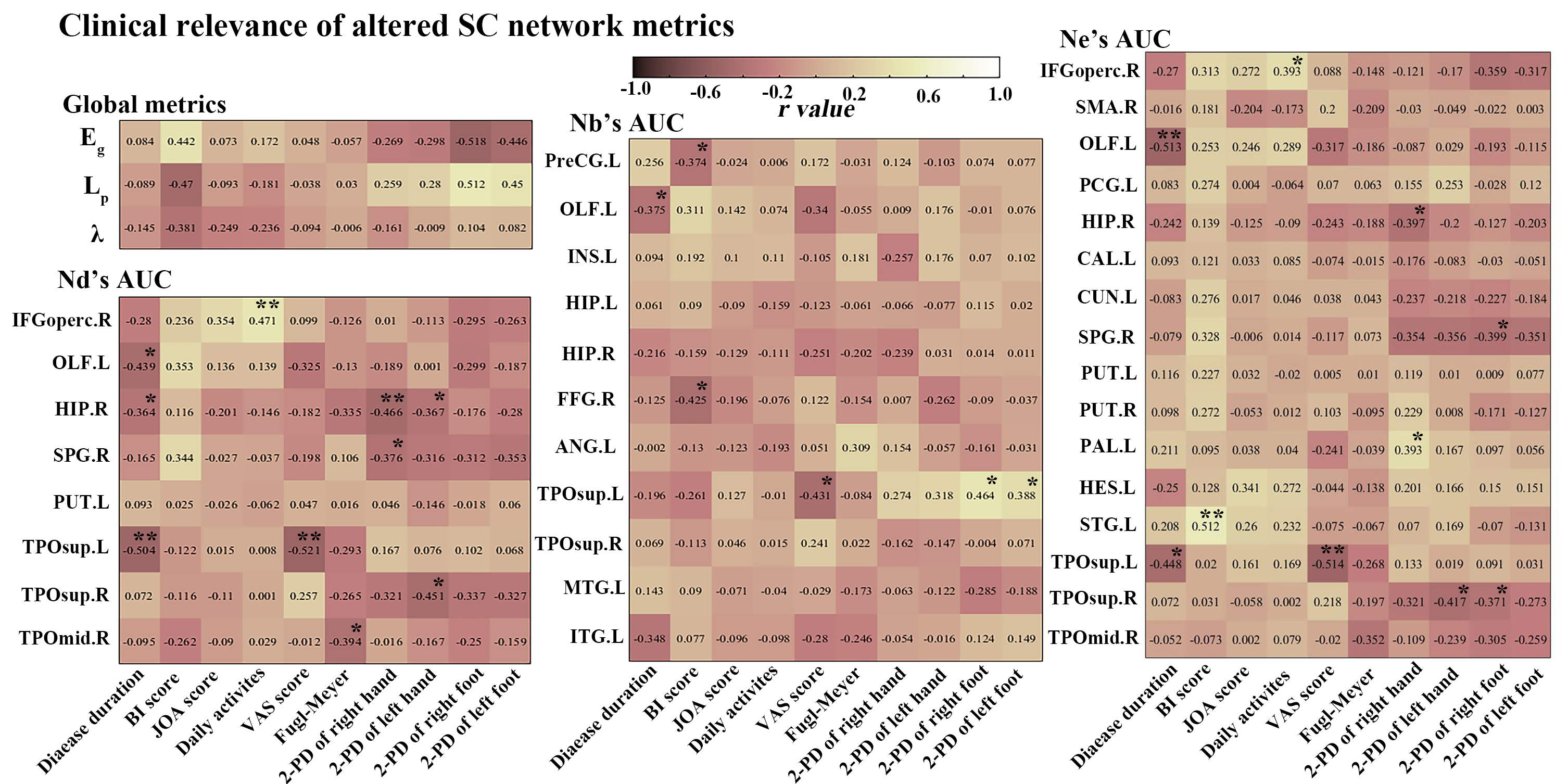

Supplement: Supplementary file 1 — Supplementary Material 1 [file 41598_2025_91570_MOESM1_ESM.tif]

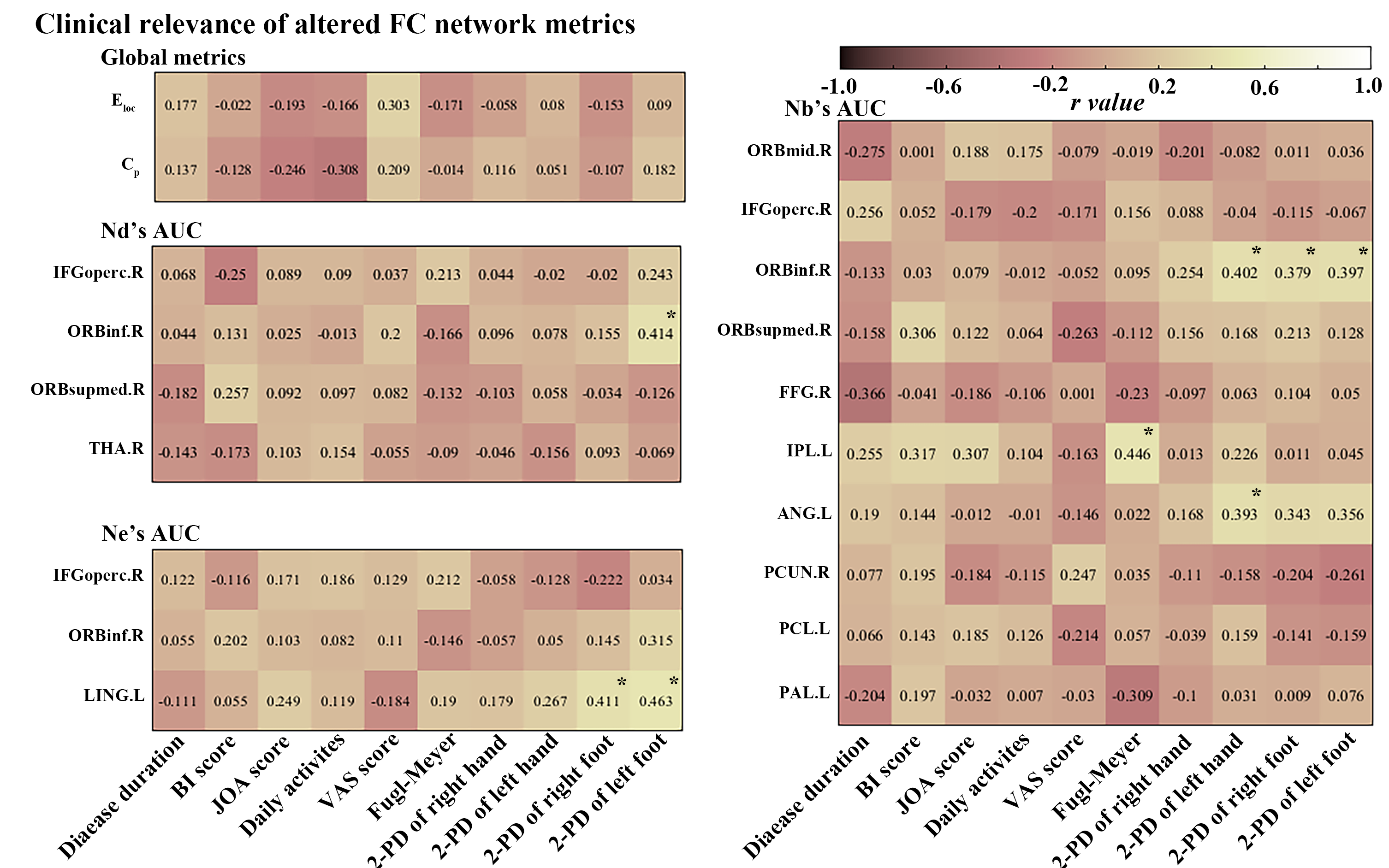

Supplement: Supplementary file 2 — Supplementary Material 2 [file 41598_2025_91570_MOESM2_ESM.tif]

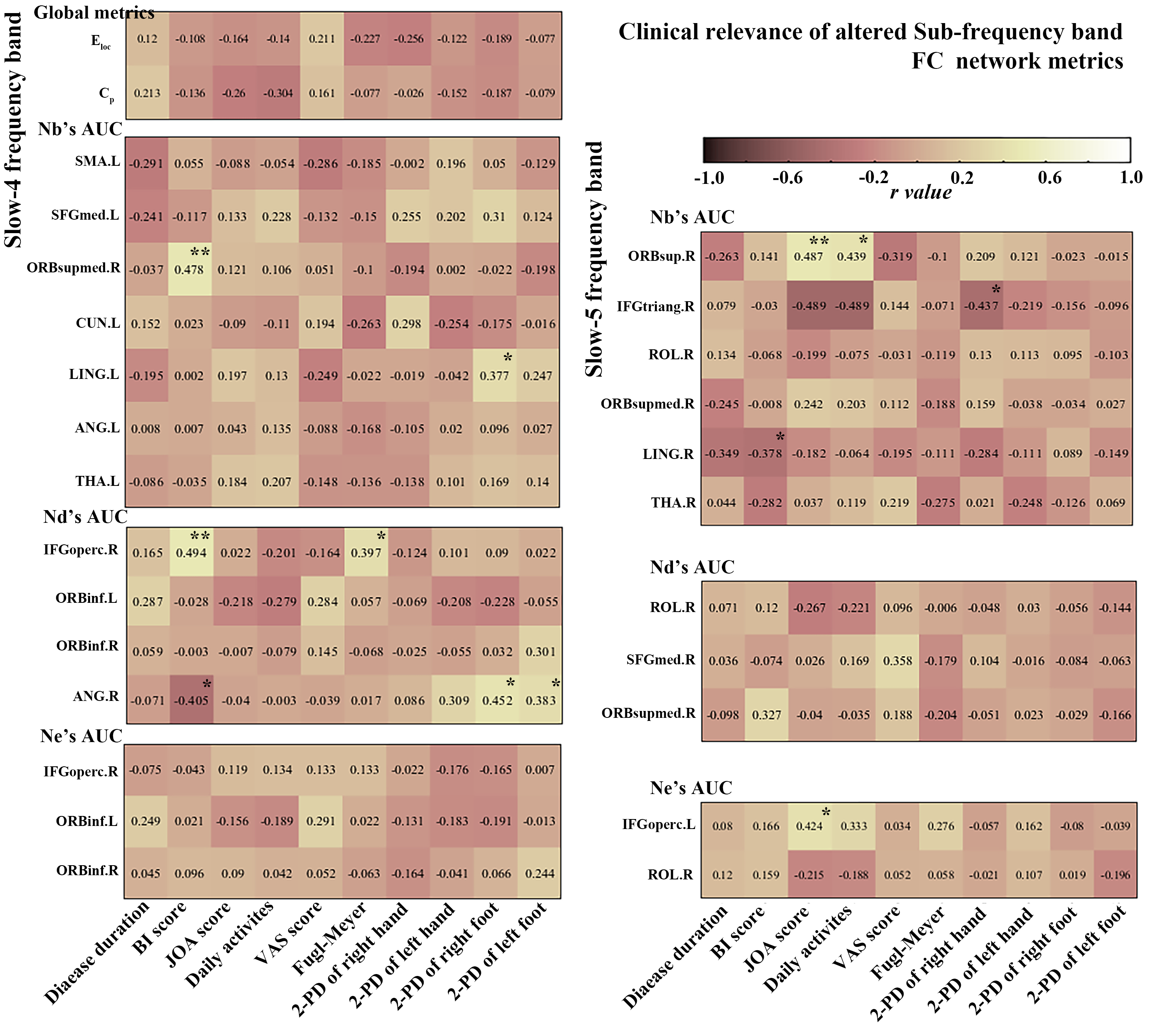

Supplement: Supplementary file 3 — Supplementary Material 3 [file 41598_2025_91570_MOESM3_ESM.tif]
