# Supplementary material for: Disrupted topological organization of brain connectome in patients with chronic low back related leg pain and clinical correlations
Source: Sci Rep. 2025 Mar 4;15:7515. doi: 10.1038/s41598-025-91570-3 (PMC11876659; doi:10.1038/s41598-025-91570-3)
Supplement: Supplementary file 4 — Supplementary Material 4 [file 41598_2025_91570_MOESM4_ESM.docx]

**Table S1**. Group comparisons of AUC values of global properties of SC network and FC networks (including sub-frequency bands) between cLBLP and HC.

|  | SC network | | | FC network | | | | | | | | |
| --- | --- | --- | --- | --- | --- | --- | --- | --- | --- | --- | --- | --- |
|  |  |  |  | Typical band | | | Slow5 band | | | Slow4 band | | |
|  | cLBLP | HC | *p* value | cLBLP | HC | *p* value | cLBLP | HC | *p* value | cLBLP | HC | p value |
| L_P_ | 0.500±0.017 | 0.561±0.021 | 0.026^*^ | 0.451±0.035 | 0.451±0.025 | 0.974 | 0.446±0.032 | 0.453±0.031 | 0.417 | 0.448±0.035 | 0.446±0.026 | 0.924 |
| C_P_ | 0.107±0.004 | 0.106±0.005 | 0.783 | 0.130±0.010 | 0.135±0.009 | 0.032* | 0.127±0.014 | 0.133±0.010 | 0.090 | 0.126±0.011 | 0.132±0.010 | 0.030* |
| γ | 0.811±0.088 | 0.848±0.074 | 0.095 | 0.378±0.056 | 0.384±0.046 | 0.644 | 0.392±0.055 | 0.398±0.058 | 0.563 | 0.376±0.058 | 0.387±0.052 | 0.399 |
| λ | 0.266±0.003 | 0.268±0.003 | 0.010^*^ | 0.254±0.005 | 0.255±0.006 | 0.848 | 0.255±0.007 | 0.256±0.006 | 0.600 | 0.253±0.006 | 0.254±0.005 | 0.624 |
| σ | 0.731±0.074 | 0.757±0.062 | 0.149 | 0.356±0.057 | 0.359±0.042 | 0.740 | 0.368±0.058 | 0.371±0.059 | 0.685 | 0.356±0.060 | 0.365±0.050 | 0.512 |
| E_g_ | 0.105±0.003 | 0.103±0.003 | 0.029^*^ | 0.131±0.007 | 0.130±0.006 | 0.701 | 0.133±0.007 | 0.131±0.008 | 0.306 | 0.132±0.008 | 0.132±0.006 | 0.828 |
| E_loc_ | 0.160±0.004 | 0.158±0.006 | 0.112 | 0.173±0,006 | 0.178±0.006 | 0.008** | 0.173±0.006 | 0.176±0.005 | 0.070 | 0.170±0.006 | 0.175±0.007 | 0.005** |

Note: Permutation tests were used to determine the differences in the AUC of global network properties; * *p* < 0.05, ** *p* < 0.01.

Abbreviations: FC, functional connectivity; SC, structural connectivity; $C_{p}$, clustering coefficient; $L_{p}$, characteristic path length; $\gamma$, normalized clustering coefficient; $\lambda$, normalized characteristic path length; $\sigma$, small-worldness; $E_{glob}$, network global efficiency; $E_{loc}$, local efficiency. The same abbreviations are used in the other figures and tables; therefore, this note is not repeated.

**Table S2.** Comparison of nodal properties of SC network between cLBLP patients and HCs.

| Brain regions | BA | P values | | | | | Yale Networks | AAL | |
| --- | --- | --- | --- | --- | --- | --- | --- | --- | --- |
|  |  | Nodal betweenness | Nodal degree | Nodal efficiency | | |  |  |  |
| **cLBLP > HC** | | | | | | | | | |
| Right IFGoperc | 44 | 0.076 | 0.020* | | 0.002** | Fronto-parietal network | | Frontal_Inf_Oper_R (12) | |
| Right SMA | 6 | 0.268 | 0.076 | | 0.022* | Limbic network | | Supp_Motor_Area_R (20) | |
| Left PCG | 23 | 0.121 | 0.545 | | 0.047* | DMN | | Cingulum_Post_L (35) | |
| Left CAL | 17 | 0.306 | 0.115 | | 0.041* | Visual I network | | Calcarine_L (43) | |
| Left CUN | 18 | 0.312 | 0.449 | | 0.038* | Visual I network | | Cuneus_L (45) | |
| Right SPG | 7 | 0.054 | 0.049* | | 0.038* | Visual association network | | Parietal_Sup_R (60) | |
| Left PUT | 49 | 0.150 | 0.001** | | 0.006** | Basal ganglia network | | Putamen_L (73) | |
| Right PUT | 49 | 0.579 | 0.083 | | 0.048* | Basal ganglia network | | Putamen_R (74) | |
| Left PAL | 51 | 0.598 | 0.067 | | 0.010* | Basal ganglia network | | Pallidum_L (75) | |
| Left HES | 41 | 0.975 | 0.236 | | 0.021* | Motor network | | Heschl_L (79) | |
| Left STG | 22 | 0.792 | 0.076 | | 0.025* | Motor network | | Temporal_Sup_L (81) | |
| Left TPOsup | 38 | 0.011* | <0.001*** | | <0.001*** | Motor network | | Temporal_Pole_Sup_L (83) | |
| Right TPOsup | 38 | 0.010* | <0.001*** | | <0.001*** | Medial frontal network | | Temporal_Pole_Sup_R (84) | |
| Right TPOmid | 38 | 0.344 | 0.009** | | 0.001** | Medial frontal network | | Temporal_Pole_Mid_R (88) | |
| **cLBLP < HC** | | | | | | | | | |
| Left PreCG | 6 | 0.034* | 0.319 | 0.698 | | Motor network | | | Precentral_L (1) |
| Left OLF | 25 | <0.001*** | 0.001** | 0.018* | | Basal ganglia network | | | Olfactory_L (21) |
| Left INS | 13 | 0.011* | 0.476 | 0.804 | | Limbic network | | | Insula_L (29) |
| Left HIP | 54 | 0.016* | 0.657 | 0.633 | | Basal ganglia network | | | Hippocampus_L (37) |
| Right HIP | 54 | 0.003** | 0.011* | 0.024* | | Basal ganglia network | | | Hippocampus_R (38) |
| Right FFG | 37 | 0.037* | 0.491 | 0.447 | | Visual association network | | | Fusiform_R (56) |
| Left ANG | 39 | 0.037* | 0.160 | 0.298 | | Fronto-parietal network | | | Angular_L (65) |
| Left MTG | 21 | 0.036* | 0.788 | 0.910 | | Medial frontal network | | | Temporal_Mid_L (85) |
| Left ITG | 20 | 0.045* | 0.438 | 0.622 | | Fronto-parietal network | | | Temporal_Inf_L (89) |

Note:  After Benjamini‐Hochberg false discovery rate corrected. All nodes have at least one type of significant nodal property change. * *P* < 0.05, ***P* < 0.01, ****P* < 0.001. All the brain regions were defined by AAL atlas. Yale Networks: Networks defined on the Shen 268 atlas.

Abbreviations: AAL, Automated anatomical atlas; BA, Brodman areas; DMN, Default mode network.

**Table S3.** Comparison of nodal properties of FC network of Typical frequency band between cLBLP patients and HCs.

| Brain regions | BA | P values | | | | Yale Networks | AAL |
| --- | --- | --- | --- | --- | --- | --- | --- |
|  |  | Nodal betweenness | Nodal degree | Nodal efficiency | |  |  |
| **cLBLP > HC** | | | | | | | |
| Right ORBmid. | 10 | 0.006** | 0.531 | | 0.558 | Fronto-parietal network | Frontal_Mid_Orb_R (10) |
| Rirht IFGoperc | 44 | 0.038* | 0.020* | | 0.011* | Fronto-parietal network | Frontal_Inf_Oper_R (12) |
| Right ORBinf | 47 | 0.041* | 0.020* | | 0.036* | Fronto-parietal network | Frontal_Inf_Orb_R (16) |
| Left LING | 18 | 0.226 | 0.070 | | 0.031* | Visual I network | Lingual_L (47) |
| Left IPL | 40 | 0.044*- | 0.783 | | 0.585 | Fronto-parietal network | Parietal_Inf_L (61) |
| Left ANG | 39 | 0.031* | 0.757 | | 0.805 | Fronto-parietal network | Angular_L (65) |
| Right PCUN | 7 | 0.049* | 0.398 | | 0.362 | Limbic network | Precuneus_R (68) |
| Left PCL | 4 | 0.037* | 0.285 | | 0.178 | Motor network | Paracentral_Lobule_L (69) |
| Left PAL | 51 | 0.043* | 0.248 | | 0.595 | Basal ganglia network | Pallidum_L (75) |
| Right THA | 50 | 0.394 | 0.047* | | 0.287 | Basal ganglia network | Thalamus_R (78) |
| **cLBLP < HC** | | | | | | | |
| Right ORBsupmed | 10 | 0.019* | 0.047* | 0.134 | | DMN | Frontal_Med_Orb_R (26) |
| Right FFG | 37 | 0.015* | 0.609 | 0.661 | | Visual association network | Fusiform_R (56) |

Note:  After Benjamini‐Hochberg false discovery rate corrected. All nodes have at least one type of significant nodal property change. * *P* < 0.05, ***P* < 0.01, ****P* < 0.001. All the brain regions were defined by AAL atlas. Yale Networks: Networks defined on the Shen 268 atlas.

Abbreviations: AAL, Automated anatomical atlas; BA, Brodman areas; DMN, Default mode network.

**Table S4.** Comparison of nodal properties of FC network of Slow4 band between cLBLP patients and HCs.

| Brain regions | BA | P values | | | | Yale Networks | AAL |
| --- | --- | --- | --- | --- | --- | --- | --- |
|  |  | Nodal betweenness | Nodal degree | Nodal efficiency | |  |  |
| **cLBLP > HC** | | | | | | | |
| Right IFGoperc | 10 | 0.544 | 0.023* | | 0.019* | Fronto-parietal network | Frontal_Mid_Orb_R (10) |
| Left ORBinf | 47 | 0.669 | 0.037* | | 0.039* | Medial frontal network | Frontal_Inf_Orb_L (15) |
| Right ORBinf | 47 | 0.085 | 0.035* | | 0.038* | Fronto-parietal network | Frontal_Inf_Orb_R (16) |
| Left CUN | 18 | 0.032* | 0.872 | | 0.528 | Visual I network | Cuneus_L (45) |
| Left LING | 18 | 0.039* | 0.037* | | 0.087 | Visual I network | Lingual_L (47) |
| Left ANG | 39 | 0.028* | 0.035* | | 0.909 | Fronto-parietal network | Angular_L (65) |
| **cLBLP < HC** | | | | | | | |
| Left SMA | 6 | 0.018* | 0.335 | 0.548 | | Motor network | Supp_Motor_Area_L (19) |
| Left SFGmed | 10 | 0.031* | 0.319 | 0.384 | | Medial frontal network | Frontal_Sup_Medial_L (23) |
| Right ORBsupmed | 10 | 0.026* | 0.161 | 0.397 | | DMN | Frontal_Med_Orb_R (26) |
| Right ANG | 39 | 0.308 | 0.049* | 0.073 | | Fronto-parietal network | Angular_R (66) |
| Left THA | 50 | 0.049* | 0.097 | 0.253 | | Basal ganglia network | Thalamus_L (77) |

Note:  After Benjamini‐Hochberg false discovery rate corrected. All nodes have at least one type of significant nodal property change. * *P* < 0.05, ***P* < 0.01, ****P* < 0.001. All the brain regions were defined by AAL atlas. Yale Networks: Networks defined on the Shen 268 atlas.

Abbreviations: AAL, Automated anatomical atlas; BA, Brodman areas; DMN, Default mode network.

**Table S5.** Comparison of nodal properties of FC network of Slow5 frequency band between cLBLP patients and HCs.

| Brain regions | BA | P values | | | | | Yale Networks | AAL |
| --- | --- | --- | --- | --- | --- | --- | --- | --- |
|  |  | Nodal betweenness | | Nodal degree | Nodal efficiency | |  |  |
| **cLBLP > HC** | | | | | | | | |
| Left IFGoperc | 44 | 0.852 | 0.103 | | | 0.030* | Medial frontal network | Frontal_Inf_Oper_L (11) |
| Right IFGtriang | 45 | 0.005* | 0.423 | | | 0.261 | Fronto-parietal network | Frontal_Inf_Tri_R (14) |
| Left THA | 50 | 0.044* | 0.693 | | | - | Basal ganglia network | Thalamus_L (77) |
| **cLBLP < HC** | | | | | | | | |
| Right ORBsup | 11 | 0.006** | 0.130 | | 0.224 | | Fronto-parietal network | Frontal_Sup_Orb_R (6) |
| Right ROL | 6 | 0.038* | 0.001** | | 0.015* | | Motor network | Rolandic_Oper_R (18) |
| Right SFGmed | 10 | 0.112 | 0.049* | | 0.139 | | Medical frontal network | Frontal_Sup_Medial_R (24) |
| Right ORBsupmed | 10 | 0.017* | 0.019* | | 0.084 | | DMN | Frontal_Med_Orb_R (26) |
| Right LING | 18 | 0.003** | 0.882 | | 0.659 | | Visual I network | Lingual_R (48) |

Note:  After Benjamini‐Hochberg false discovery rate corrected. All nodes have at least one type of significant nodal property change. * *P* < 0.05, ***P* < 0.01, ****P* < 0.001. All the brain regions were defined by AAL atlas. Yale Networks: Networks defined on the Shen 268 atlas.

Abbreviations: AAL, Automated anatomical atlas; BA, Brodman areas; DMN, Default mode network.

**Figure S1.** Correlations between SC network metrics and clinical variables in patients with cLBLP.

Note: * *P* < 0.05, ***P* < 0.01 without false discovery rate (FDR) correction.

**Figure S2.** Correlations between FC network metrics (in the typical frequency band) and clinical variables in patients with cLBLP.

Note: * *P* < 0.05, ***P* < 0.01 without false discovery rate (FDR) correction.

**Figure S3.** Correlations between the FC network metrics of slow-4 and slow-5 frequency bands and clinical variables in patients with cLBLP.

Note: * *P* < 0.05, ***P* < 0.01 without false discovery rate (FDR) correction.
